# Central nervous system control of breathing in natural conversation turn-taking

**DOI:** 10.1038/s41598-025-15776-1

**Published:** 2025-08-25

**Authors:** Camilla Di Pasquasio, Lila De Pellegrin, Arthur Pineaud, Antonin Marty, Thierry Chaminade

**Affiliations:** Institut de Neurosciences de la Timone UMR 7289 Aix-Marseille Université - CNRS, 25 Bd Jean Moulin, 13385 Marseille cedex 5, France

**Keywords:** Speech breathing, Human communication, Conversational turn-taking, Respiration, fMRI, Social interaction, Respiratory control, Central sulcus, Brainstem, Cerebellum, Language, Respiration

## Abstract

Conversations—hallmarks of human social interaction—rely on a deeply rooted evolutionary tool: vocalization. Vocalization productions begin with an airflow from the lungs, shaped by the vocal folds and articulators of the mouth to generate a rich variety of sounds. Speech production therefore involves tight synchronisation between respiratory and articulatory events, especially in natural social interactions. During conversations, turn-taking indeed implies respiration anticipation: lungs must be filled with enough air to produce the next speech segment. Here, we use an fMRI corpus of live, unconstrained conversations to identify key brain regions involved in the respiratory anticipation of speech onset. Behaviourally, respiratory local maxima preceded speech onset (“Resp+” events) by approximately 200 ms, consistent with volitional control of breathing overriding automatic respiratory rhythms. Contrasting Resp+ events with respiratory maxima not linked to speech onsets (“Resp−” events) revealed significant (*p*_*FWE*_ < 0.05, k > 5 cm³) bilateral responses in the postcentral sulcus, brainstem, and cerebellum. Brainstem respiratory control nuclei are known to generate automatic breathing rhythms while integrating multimodal signals for gas exchange homeostasis. The postcentral sulcus has been implicated in processing afferent feedback from respiratory effectors during intentional breathing, and the cerebellum maintains reciprocal anatomical connections with both brainstem respiratory centres and cortical respiratory control areas. Together, these structures form a network that inhibits automatic respiration for volitional respiratory control of speech onset. Our findings underscore the interaction between cortical and subcortical regions involved in respiratory control for speech production in social interactions, and offer new insights into the neural foundations of human communication and its disorders.

**Supplementary Information:**

The online version contains supplementary material available at 10.1038/s41598-025-15776-1.

## Introduction

 Language is a unique hallmark of human cognition, and conversations, from everyday chit-chat to complex discussions, play a widely acknowledged role in social interactions^1^. Before speaking, humans typically take a short, rapid inhalation. Though seemingly simple, this act reflects a sophisticated integration of automatic and voluntary control over breathing. Indeed, breathing is unique among autonomic functions: while vital rhythms like heartbeat or digestion remain involuntary, respiration is modulated both reflexively, via brainstem centres that sustain life even during sleep or anaesthesia, and voluntarily, through cortical pathways that adapt breathing to behavioural demands. Speech production, in particular, requires top-down cortical control to override metabolic rhythms in order to synchronize inhalations with conversational turns. This dual respiration control, automatic and volitionary driven, requires coordination between brainstem structures, necessary for automatic breathing, and supratentorial areas such as the motor cortex, cerebellum, and thalamus, which have all been implicated in volitional respiratory acts such as breath-holding, speaking, and singing^2^. In conversation, this volitional override becomes especially critical, as speakers must time their inhalations to anticipate and align with the dynamics of turn-taking.

While prior work has suggested that respiration plays a role in conversational turn-taking at both physiological and behavioural levels^3–5^, the neurophysiological mechanisms underlying this integration remain largely unknown. Since speech breathing involves both volitional and automatic control mechanisms in the brain^6^, it provides a unique window into how the brain integrates physiological functions with communicative goals. To understand this integration, it is crucial to distinguish between breathing that serves metabolic needs and breathing that is coordinated with speech. Disentangling these two modes of respiration in the context of natural conversation is therefore essential for advancing models of sensorimotor integration and the neurobiology of social interaction. Here, we address this question by combining functional magnetic resonance imaging (fMRI), respiratory signals, and speech transcripts from 25 participants engaged in live unconstrained conversations with human and robotic interlocutors. We identify responses in cortical and subcortical regions involved in the timing and modulation of respiration control, contributing novel insights into the neurobiology of conversational turn-taking.

### Conversation, a social implementation of speech

Understanding how respiration and speech are integrated is essential because speaking requires precise modulation of breathing, emerging from the interplay between involuntary brainstem-driven rhythms and volitional cortical control. Moreover, the respiratory system adapts dynamically to communicative demands, and inspirations intended for speech are temporally and functionally distinct from those driven by metabolic needs^6^. An important aspect of this adaptation is the temporal alignment of breathing across interlocutors, referred to as “conversational” breathing, that reflects a broader sensorimotor coupling that supports verbal interaction^1^. To understand how respiration and speech are integrated in conversation, it is essential to consider how turn-taking relies on precise temporal coordination between speakers^3^. Conversation consists of spontaneous spoken exchanges where speakers alternate rapidly, often within a few hundred milliseconds^6^. This swift coordination is not only a linguistic feat, but also a physiological one: to speak, individuals must produce phonatory exhalations while also timing their breathing with the ongoing flow of dialogue. Successful turn transitions between interlocutors require accurate anticipation of when a speaker will yield the floor, enabling the next speaker to prepare their response in advance (i.e., turn-taking). This anticipatory ability enables efficient communication by reducing both pauses and overlap, a pattern observed across languages and cultures^4^. Beyond language processing, respiratory cycles are key in regulating this interaction: speakers often time their inhalations to coincide with the end of their interlocutor’s turn, thus establishing a tight temporal link between respiration and speech planning^1,5^. Behavioural evidence further suggests that speech-related breathing follows asymmetrical cycles, with short, rapid inhalations (200–500 ms) and prolonged exhalations aligned with speech production periods^3^. Respiratory pauses within utterances tend to be shorter than those preceding new speech segments, revealing subtle modulations for conversational fluency. Failed interruptions—when a listener attempts to speak while the current speaker continues—often occur in the absence of preparatory inhalation cues, underscoring the importance of respiratory readiness for successful turn-taking^3^. Similarly, successful turns tend to begin at the onset of the exhalation phase, further illustrating how physiological rhythms scaffold conversational dynamics^3,7^. This highlights the need to investigate this coordination while taking into account sources of variability^8^. Notably, respiratory coordination extends beyond the speaker. Listeners also adjust their breathing patterns in response to conversational flow. Increased respiratory rate and reduced inspiration duration are observed in anticipation of turn-taking, indicating a bidirectional coupling of respiration between interlocutors^3,7^. This synchronization reflects an implicit alignment of respiratory dynamics within social interaction^9^.

### Central nervous system control of speech

This coordination involves complex physiological processes, particularly in the neural control of respiration and articulation effectors. Research suggests a dissociation between the dorsal and ventral motor cortices in human speech, corresponding to the control of respiration and articulation, respectively^2,10–13^.

The neural bases of speech articulation have been extensively studied through neuroimaging and lesion-symptom mapping. These investigations have revealed a distributed network that includes the inferior frontal gyrus, premotor and motor cortices, insula, supplementary motor area, and subcortical structures. This network supports articulatory planning, motor execution, and coordination with respiration during speech production^13^.

By contrast, little is known about the neurophysiological control of breathing in speech, despite its importance, particularly in conversations.

While the central nervous system’s ability to control respiration in natural conversations remains unknown, the neuropsychological literature reports a significant number of cases of dissociation between automatic and voluntary breathing. A patient study on a case of severe respiratory apraxia resulting from supranuclear palsy revealed an inhibition of automatic breathing upon volitional control^14^. Ventilation compensation by the supplementary motor area (SMA) during arousal is suggested in patients with congenital central hypoventilation syndrome^15^. Both examples illustrate a complex interplay between volitional and automatic control of respiration, the former being able to temporarily bypass the latter, a unique case for a life-sustaining physiological requirement in animals^16^. Research in animals, further supported by human neuroimaging investigations of volitional respiratory control^2,17,18^ and a model of dyspnoea^19^, suggest a complex interplay between respiration-related cortical networks and the involvement of automatic respiration generation in the brainstem.

In particular, a brainstem respiration controller consists of a network of respiratory neurons projecting to motoneurons in the spinal cord located in the ventral reticular formation, which are innervating the ribcage and upper airways^20^. The primary circuits responsible for generating respiratory rhythms are located within the medullary ventral respiratory column (VRC) and are typically categorised into distinct functional and chemical subregions, including the retrotrapezoid nucleus/parafacial respiratory group (RTN/pFRG)^21^, postinspiratory complex (PiCo)^22^, Bötzinger complex (BötC)^23^, preBötzinger complex (preBötC)^24^, and rostral and caudal ventral respiratory groups^25^. Extensive animal studies have led to the identification of respiratory pattern generators, known collectively as the preBötzinger Complex [preBötC,^26^], as central elements of this controller.

This preBötC orchestrates the respiratory cycle phases, primarily focusing on inspiration, while also integrating aspects of post inspiration and expiration^27^. Although it is primarily responsible for inspiration, the Postinspiratory Complex (PiCo) plays a role in expiration and integrates information essential for controlling the dynamics of both inspiration and expiration. Because the brainstem primarily regulates automatic breathing for survival, its role in volitional control of respiration—particularly for speech—remains unclear. Breathing is a unique physiological function that is largely automatic but can also be voluntarily controlled^2,12^. In conversational speech, respiration must synchronize with expiration, potentially leading to conflicts between automatic and volitional control mechanisms^3,6^. Understanding how cortical, cerebellar and brainstem networks coordinate breathing in natural conversation is crucial to delineate how respiration serves both survival and communication.

### The current study

Building on the notion that respiration supports both survival and communication, the present study aimed to disentangle the neural mechanisms that govern these distinct breathing functions during real-life verbal interaction. While previous research has explored respiratory control in isolated or task-based contexts, little is known about how the brain coordinates breathing in spontaneous conversation. Our aim is to identify key neural areas involved in the control of breathing by the brain according to whether it is associated with speech production (maximum occurrence close to the participants’ speech onset) or physiological survival (necessity of pulmonary exchange between O_2_ and CO_2_).

To address this issue, we made use of a unique corpus of natural conversations comprising synchronised conversation, breathing, and functional neuroimaging (fMRI) data. This dataset allowed us to examine how breathing patterns differ depending on whether they are temporally aligned with speech onset or not—an approach that leverages the continuous nature of both the respiratory signal and conversational flow. Rather than testing a priori hypotheses, our aim was to characterize how different brain regions contribute to the modulation of breathing during spontaneous speech. By distinguishing between breathing events linked to speech initiation and those likely driven by metabolic needs, we sought to identify specific neural circuits involved in conversational breathing. After synchronizing speech transcripts with the respiration recordings, we aim to identify which cortical and subcortical structures are involved in modulating breathing in anticipation of speech production. To do so, we categorized respiration maxima as related or unrelated to speech based on their temporal proximity to speech onset, enabling us to distinguish speech breathing from respiration driven by metabolic needs.

## Materials and methods

### Corpus acquisition

Twenty-five native French-speaking participants (7 M, 18 F) with an average age of 28.5 years (*s.d.* 12.4) were recorded in an fMRI protocol investigating natural conversations in human–human and human–robot interactions. The experimental procedures were approved by the designated national ethical committee (“Comité de Protection des Personnes” CPP Sud-Marseille 1, approval number 2016-A01008-43), and informed consent was obtained from all participants after they were fully informed about the experimental procedures. The methods were performed in accordance with all relevant guidelines and regulations. The conversational paradigm, its theoretical grounds^28^, and the variables recorded^28^ have already been described^28,29^, and we will focus on the three variables utilised in the current analysis: brain activity (using fMRI blood oxygen level-dependent (BOLD) signals acquired continuously), respiration (focusing on inspiration-to-expiration transitions) recorded with an abdominal respiration belt, and transcripts of the natural conversations (interest in the presence or absence of speech).

The participants were fully naive to the actual objectives of the current study, i.e., creating a multidimensional corpus of natural interactions, including synchronised neurophysiological recordings. At the MRI centre, they were presented with a cover story, which involved a natural discussion about a purported neuromarketing experiment, and installed in the scanner. A bidirectional audio setup enabling live conversation between the participant in the scanner and an interlocutor located outside consisted of an active noise-cancelling MR-compatible microphone (FORMI/III + Optoacoustics Ltd., https://www.optoacoustics.com/medical/fomri-iii) to reduce scanner noise and earphones from Sensimetrics inserted in participants’ ears. Participants viewed a live video feed of the interlocutor projected onto the scanner’s stimulation screen. The interlocutor was either a confederate of the experimenter (human) or the robot, a conversational head-shaped robot equipped with speech synthesis with lips synchronized and facial animations (Furhat robotics, https://www.furhatrobotics.com/). Furhat was operated by a human confederate, who used around 100 scripted sentences prepared in advance. This setup, known as the Wizard of Oz flamework^30^, makes the robot appear to converse independently.

Four fMRI sessions of whole brain recordings were acquired, -. During each session, participants took part in six live conversations trials — alternating between the human and the robot interlocutor. Each trial began with the presentation of one of three images used to prompt discussion, followed by one minute of live unconstrained conversation with the interlocutor. Four sessions of 6 1-minute conversations yielded an overall total of twenty-four minutes of conversation recorded per participant, for a concatenated total of 10 h of conversation recorded for the corpus of 25 participants.

The MRI data were collected with a 3 T Siemens Prisma (Siemens Medical, https://www.siemens-healthineers.com/it/magnetic-resonance-imaging/3t-mri-scanner) using a 20-channel head coil. BOLD-sensitive functional images were acquired via an EPI sequence in the four sessions. The parameters were as follows: echo time (TE) 30 ms, repetition time (TR) 1205 ms, flip angle 65°, 54 axial slices coplanar to the anterior/posterior commissure plane, field of view 210 mm × 210 mm, matrix size 84 × 84, and voxel size 2.5 × 2.5 × 2.5 mm^3^, with a multiband acquisition factor of 3. After functional scanning, structural images were acquired with a GR_IR sequence (TE/TR 0.00228/2.4 ms, 320 sagittal slices, voxel size 0.8 × 0.8 × 0.8 mm^3^, field of view 204.8 × 256 × 256 mm). Three-dimensional images of blood oxygen level-dependent (BOLD) signals were acquired via whole-brain scans every 1.205 s. One session included 385 volumes for a duration of 463.925 s. The raw data acquired during fMRI scanning have been uploaded to OpenNeuro (https://openneuro.org/datasets/ds001740*)*, including text-format log files containing trial onset information.

The study’s physiological data (cardiac pulsation and respiration) were recorded with the dedicated apparatus of the scanner. A photoplethysmography unit was positioned on the left-hand index fingertip to record pulse oximetry and a breathing belt was positioned at the chest level. Both were connected wirelessly through Bluetooth. Data was acquired continuously at the frequency of 200 Hz. Data format being proprietary, a pre-processing is needed using a specific toolbox (PhysIO Toolbox). Synchronisation is intrinsic to the data recording system and is based on the 2.5-ms time bins constitutive of MRI recordings: time is logged in bins for the BOLD signal and the breathing data, both recorded with the MRI scanner, and the conversational data processing also includes a synchronisation and resampling step (see next).

### Processing of physiological time-series

fMRI pre-processing with SPM12 (Statistical Parametric Mapping, 2014, https://www.fil.ion.ucl.ac.uk/spm/software/spm12/) involved slice-timing correction, volume realignment and magnetic field inhomogeneity correction^31^. Normalisation of individual functional and anatomical data to the standard brain space of the Montreal Neurological Institute (MNI) used segmented anatomical T1 and T2 images recorded just after BOLD functional images and realigned with the first image of the time series, followed by the DARTEL procedure^32^.

The CONN toolbox (CONN functional connectivity toolbox, version 13, https://web.conn-toolbox.org/*)* was used to compute nuisance regressors for denoising the BOLD signal for the individual participants’ level of analysis^33^. Linear detrending was applied using a high-pass filter with a cut-off of 128 s. Realignment parameters were included to account for artifacts related to participant head motion during scanning. Additionally, signals extracted from whole-brain masks corresponding to grey matter, white matter, and cerebrospinal fluid—identified from invidiual participant’s segmented anatomical image—were used to model physiological fluctuations in the BOLD signal that were unrelated to the experimental manipulation.

Physiological recordings were processed via the PhysIO Toolbox (TAPAS PhysIO Toolbox,version9.0.1,https://github.com/translationalneuromodeling/tapas/blob/master/PhysIO/README.md)^34^, which employs model-based approaches for physiological noise correction of fMRI data. It uses the raw physiological time-series to derive heart rate variability (referred to as the cardiac response function) and respiratory volume (referred to as the respiratory response function). Both movement-related and physiology-related possible sources of artefacts were exported as single-participant and single-session matrices containing aggregating cardiac, respiration, and movement signals possibly associated with recording artefacts as well as linear and region-of-interest detrending nuisance variables. Finally, we applied 5 mm^3^ full-width half-maximum three-dimensional Gaussian kernel spatial smoothing.

Saturation in the respiratory belt signal was identified in time-series as flat segments—plateaus—where identical extreme values persisted across consecutive time bins, typically during strong inspirations. To address this, all time-series were first smoothed using a 50-bin (250 ms) moving average to facilitate the detection of these saturated intervals. They were then automatically flagged as having null derivative in the smoothed signal. Corresponding data was treated as missing and replaced using a *spline* interpolation in in MATLAB’s *interp1* function (MathWorks, version R2022a, https://www.mathworks.com/), leveraging the full duration of each session in the process to preserve its overall temporal coherence. The final time series were resampled at millisecond resolution and temporally aligned with the fMRI acquisition, using the known synchronization between the respiration signal offset and the end of the fMRI recording session.

### Processing of behavioural time-series

Inter Pausal Units (IPUs)—defined as periods of continuous speech occurring between pauses (or silences) lasting longer than 200 milliseconds^35^—were used as the basic segmentation unit for analysing conversational timing and speech-respiration alignment. Conversational audio recordings were first denoised to reduce scanner-related artifacts using a noise reduction filter implemented in SoX (Sound eXchange, version 14.4.2, http://sox.sourceforge.net). For each participant, an individualized noise reduction coefficient (float value between 0.01 and 0.50) was applied. The cleaned audio signal was then segmented into IPUs using a participant-specific thresholding procedure. A float coefficient (0.20 to 0.95) was applied to the mean of the root mean square (RMS) distribution to automatically determine amplitude thresholds for classifying silence versus speech. These thresholds allowed for accurate detection of IPU boundaries across varied speech intensities. All resulting speech segments were imported into SPPAS (v1.9.9)^36^ for manual orthographic transcription. Transcripts were then normalized using SPPAS’s built-in automatic text normalization tools. IPU segmentation was subsequently used to align spoken utterances with respiratory cycles in the main analysis. While the speech signal was transcribed and normalized, the verbal content and linguistic features of the conversations were not analysed in the present study. Our focus was limited to the timing and alignment of respiration and speech, rather than the linguistic structure. For more detailed analyses of linguistic aspects of the corpus, see Hallart et al.^29^.

### Definition of experimental events

Automatic processing algorithms were developed in MATLAB to (1) identify local respiration maxima corresponding to transitions between inspiration and expiration, and (2) associate each participant’s speech onset with the closest, in time, temporal respiration maximum. Combining these two sources of information, two events expected to differ in terms of neurophysiological correlates^27^ were defined: respiration maxima associated with participants’ speech onset (“Resp+”) and other respiration maxima (“Resp-“).

First, we used the log files related to fMRI recordings to identify single block onsets and then added this value to the onsets of the IPU produced by the participant in this block (i.e., the moment the participants take their turn in the conversation) to obtain the onset of each speech turn from the scanned participant across all conversation trials and for the four sessions. Respiration maxima temporally closest (in absolute value) to all single IPU onsets were categorised as *“respiration maxima associated with speech production*” (Resp+), whereas all other were attributed to “*respiration maxima not associated with speech production*” (Resp-). Attributing single Resp + events to every IPU event allowed us to calculate, for each IPU produced by the participant, the relative time difference between the IPU onset and the closest respiration maximum (Δt).

**Fig. 1:**
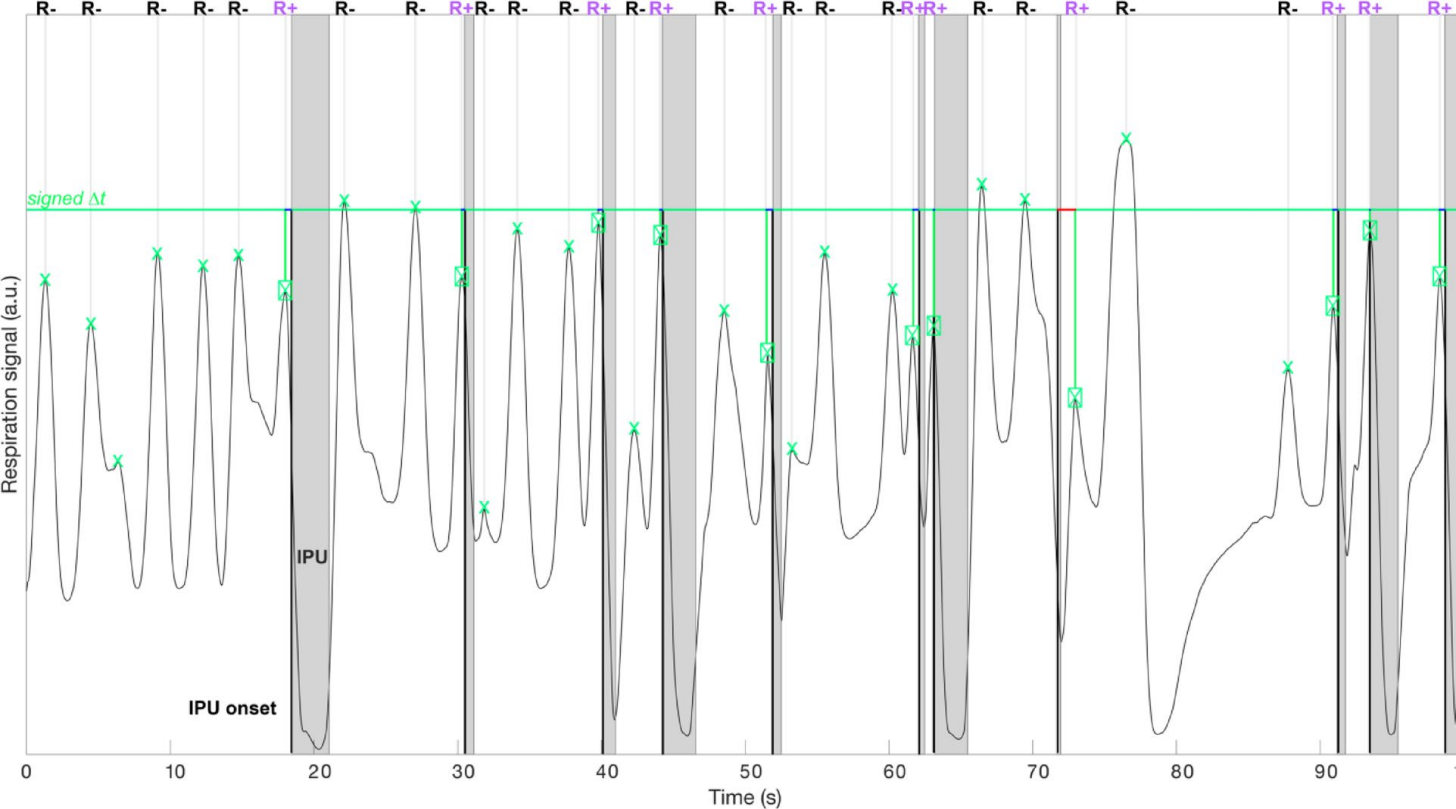
Example of respiration signal synchronized to IPUs for one participant and one session, showing (x = relative amplitude, y = time). Grey vertical bars indicate Inter-Pausal Units (IPUs), with their onsets marked by the bold black line of each shaded area. Green squared crosses represent detected respiration peaks for IPU related peaks (Resp+), simple green crosses represent respiration peaks non associated with IPUs (Resp-). For each IPU, the respiration peak closest in time to the IPU onset is classified as Resp+, while all other peaks are labeled Resp−.

These respiration events (Resp + and Resp−) are defined independently of other biomechanical characteristics and are based solely on their functional association with speech and their temporal dynamics. Note that the respiration amplitude is expressed in arbitrary units, as respiration was recorded using the MR-compatible belt in the MRI scanner. A visualisation of a respiration track synchronized with IPUs timing for one participant in one session is shown in Fig. 1 and displays the respiration signal synchronized to IPUs. The respiration signal (y-axis: relative amplitude; x-axis: time in seconds) is plotted alongside shaded grey areas marking the duration of IPUs, with their onsets indicated by bold black vertical lines. Respiration peaks were algorithmically extracted, and the peak closest in time to each IPU onset was classified as *Resp+*, reflecting respiratory events temporally aligned with the initiation of speech. These are marked as green squared crosses. All other respiration peaks, not associated with an IPU onset, were classified as Resp − and are shown as simple green crosses. The classification process was fully automated, ensuring reproducibility across participants and sessions.

### Analyses

Analyses were performed across the 4 sessions of the 25 participants included in the corpus. For the behavioural analysis, we used MATLAB’s function *rmoutliers* to identify and remove outliers from the Δt values, i.e., more than three median absolute deviations from the median. The percentage of removed data was less than 5%, allowing the use of all 25 participants and all four sessions, analysing the complete dataset only excluding outlier points. Kolmogorov-Smirnov tests of normality were run on the resulting cleaned Δt values on the final time value distribution and reached the significance level (*p* < 0.05) for most participants and sessions, indicating that the data were not normally distributed. We therefore performed skewness tests to measure the asymmetry of the distribution.

For each participant and session, BOLD data were analysed with SPM12 with three conditions, image presentation, which will not be discussed further, and the two conditions of interest, Resp + and Resp-, modelled as events (i.e., zero duration). All nuisance variables calculated with the PhysIO Toolbox were introduced in these models, allowing the removal of artefacts associated with physiological (breathing and heartbeat) noise, movement artefacts and detrending on the basis of linear as well as region-of-interest signals^34^.

Given that Resp + events are temporally synchronised with IPU onset, hindering the distinction between neurophysiological correlates of respiration maxima associated with articulatory correlates of speech onset from respiration control during speech production, we used an exclusive masking procedure to remove brain activity associated with speech production in our corpus. To do so, we applied a multi-step thresholding strategy. First, we computed the contrast with the conditions “*IPUs from participant*” (IPU+) and “*IPUs from interlocutor*” (IPU-), which described each produced and perceived speech turn, for the participants, by its onset and its duration. We computed this contrast at the second level, using the same parameters as the main analysis and thresholded at *p*_*FWE*_ < 0.001 and saved as a binary image. To prevent border effects, we expanded the mask by two voxels in all directions using MRIcron. The resulting mask, illustrated in **Supplementary Fig. 1**, included all brain areas involved in the articulatory aspects of speech, namely large parts of the bilateral sensorimotor cortex and superior temporal gyri extending to the left posterior inferior frontal cortex, as well as bilateral medial motor cortex known as Supplementary and pre-Supplementary Motor Areas (SMA and pre-SMA). An additional bilateral response in the anterior putamen is not visible in the surface render.

Next, for the second-level analysis of interest, individual beta estimates from the two conditions of interest Resp + and Resp- were used in a full ANOVA model using SPM12, with participants and sessions as repeated measures (unequal variance for the former, equal for the latter). We used the exclusive mask described previously to exclude brain regions associated with speech production. The masked analysis was thresholded at *p*_*FWE*_ < 0.05 at the peak level and *p*_*FDR*_ < 0.05 at the cluster level (minimum cluster size of k > 5 cm^3^).

### Central sulcus localisation

We conducted a single-participant and single-session analysis to identify more precisely the location of the central sulcus cluster in each hemisphere, particularly to distinguish between precentral and postcentral localisations. Significant activity (*p*_*unc*_ < 0.001) was detected within a 1 cm radius around the group analysis peak in 145 out of 200 possibilities (25 participants × 4 sessions × 2 hemispheres), i.e., almost 3/4. We developed a procedure to minimise subjective biases to localise the respective position of the peak of individual activity concerning the position of the central sulcus. Human intervention was minimised to two aspects of the procedure, and automatic MATLAB and Python (version 3.11, https://www.python.org) scripts were used to draw the central sulcus and overlay the central sulcus drawing on the activity map for each participant, session, and hemisphere in which activity was found.

We first saved an axial section of the normalised structural image made at the height of the peak of the identified cluster, usually around z = 60 mm in MNI coordinates, without any activity overlaid. Three independent observers identified the central sulcus on these images. The absence of any discrepancy between the observers validated the identification of the central sulcus on each structural section.

The images were then cropped manually with Photoshop (Adobe Photoshop, version 2022, https://www.adobe.com/products/photoshop.html) to contain only the central sulcus region identified. The tracing of the central sulcus was semiautomated via ad hoc Python scripts that drew the sulcus on the basis of the grey value intensity of the anatomical image. To bootstrap sulcus drawings, a starting point was selected manually. As the sulcus never reaches the midline at this level of the brain, we identified the pixel that could still be identified as grey matter found at the fundus of the central sulcus closest to the midline. A Python script drew a line corresponding to the central sulcus iteratively by looking for the adjacent pixel with the lowest intensity centrifugally.

Finally, a MATLAB script overlaid the line drawing corresponding to the central sulcus onto the functional image in which the peak location of the activated cluster is indicated by a cross yielding a series of images allowing us to categorise the respective location of the individual activation peaks and the central sulcus, with four possibilities: (1) the cross falls on the line, (2) at the fundus when it is close (less than one pixel) from the starting point chosen arbitrarily, and for all others, (3) anteriorly or (4) posterior to the central sulcus.

## Results

### Behavioural analysis

Altogether, a total of 21,542 respiration maxima were identified, 4313 of which (approximately 20%) were temporally closest, in absolute value, to speech onset during conversation trials.

**Fig. 2:**
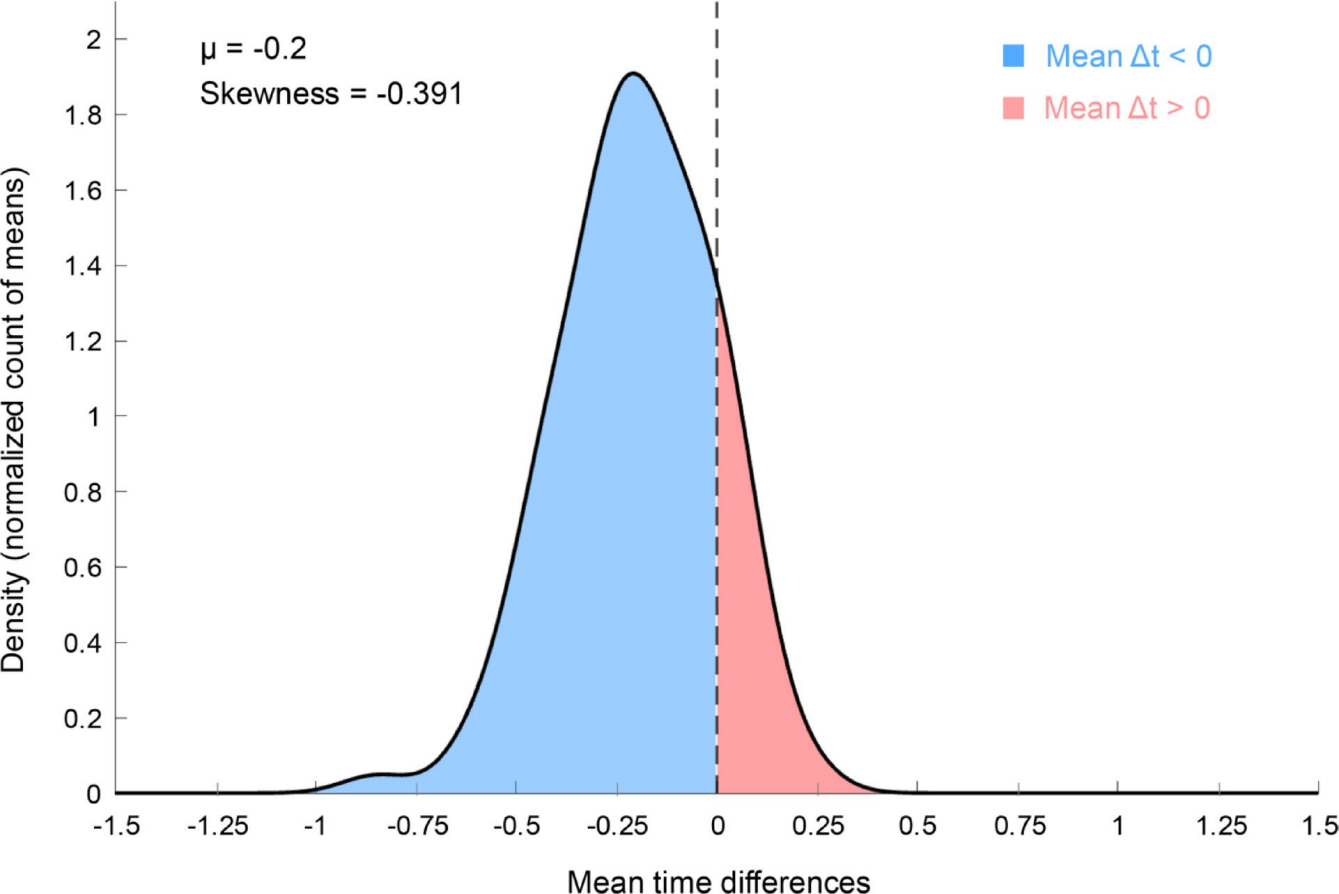
Cumulative density plot of means of time differences for all participants between IPU onset and max breathings (Δt). The plot shows a left skewness peaking at 200 ms, indicating that the maximum of breath occurred on average 200 ms preceding participants’ speech onset.

Analysis of skewness in the relative timing between Resp + events and the corresponding IPU onset Δt revealed a significant left-skewed distribution, with a maximum of approximately 200 ms of respiration preceding speech onset (−0.2003 s), as illustrated in the cumulative density plot in Fig. 2. This finding indicates that the maximum breathing intake occurred, on average, 200 ms before the participants’ speech onset. The finding of maximum breath following speech onset (in red in Fig. 2) is surprising, but it is likely due to timing errors associated with the breathing maximum estimate, as interpolation of the saturated signal could fail to reproduce some aspects of the breathing signal, such as the asymmetry between the dynamics of inspiration and expiration^8^. Additionally, the timing of IPU onset also suffers from possible timing errors depending on the type of phoneme produced at the beginning of speech production, especially given the noisy environment of the MRI scanner. Importantly, while these are intrinsic limitations of the current investigation, they are unlikely to interfere with the fMRI results given their poor temporal limitations. The statistical power of the fMRI analysis comes from the repetition of events of interest, which combine all respiration cycles recorded during one acquisition session, i.e., several hundreds of breathing maxima for approximately 7-min and 45-sec sessions, with variance in the timing between events related to other behavioural factors such as the presence or absence of speech.

A two-sample t-test was used to compare the average ∆t (IPU onset to respiratory peak) between human–human and human–robot interactions. The results (Human mean = − 0.2063 s, *s.d.* 0.1752; Robot mean = − 0.1804 s, *s.d.* 0.1617; t(7169) = 0.97, *p* = 0.34) showed no significant difference, and the distributions were highly similar (Supplementary Fig. 4). Because no significant behavioral differences were observed between human-human and human-robot interactions, we analysed the data without distinguishing between these two types of interactions. Since the current analysis focuses on speech production from the scanned participant, data from both human–human and human–robot conversations were pooled together in the analysis.

### Second-level analysis of respiration maxima related to IPUs

**Fig. 3:**
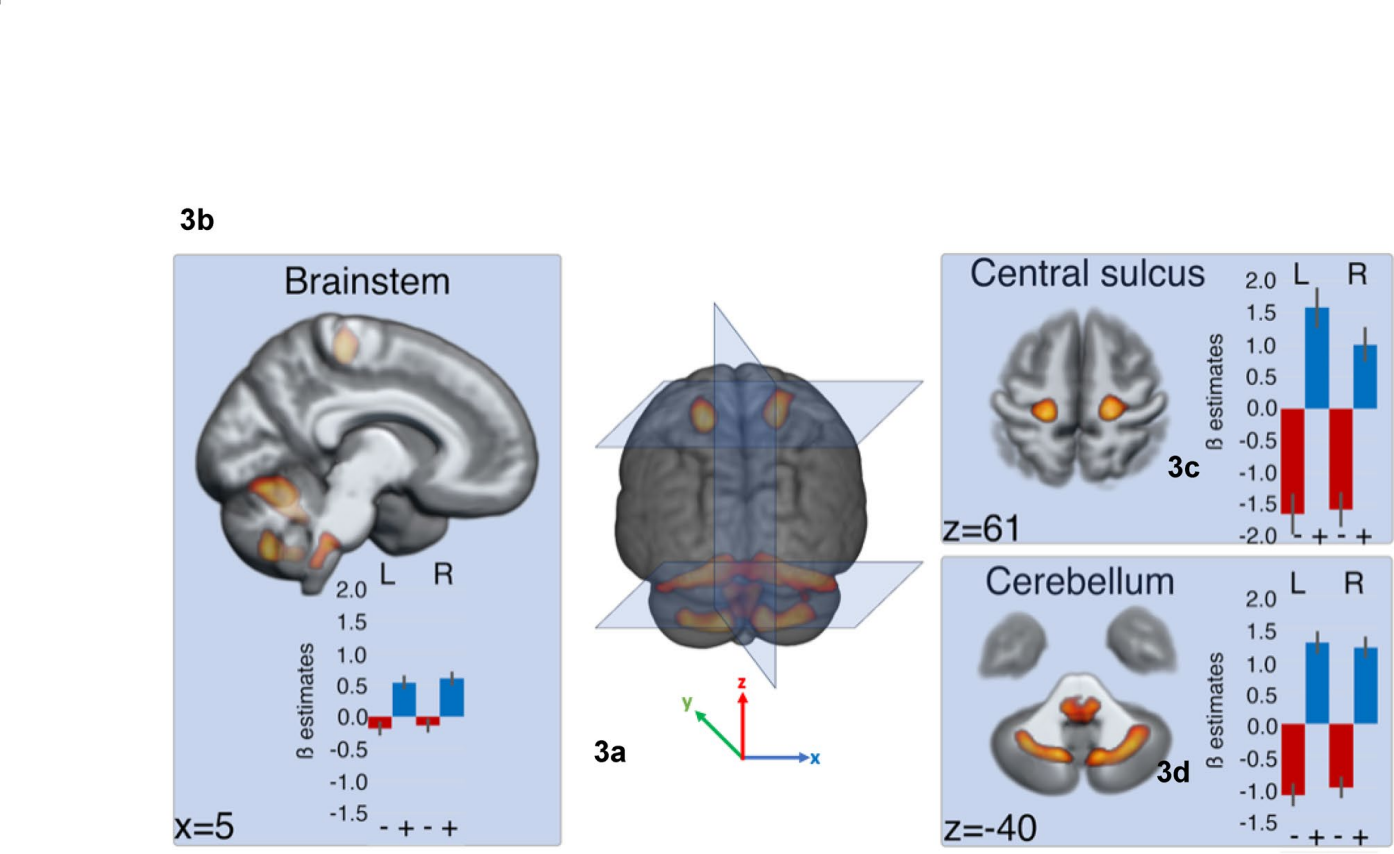
**a** Brain activations for maximum breath intake associated with a speech onset (Resp + *versus* Resp- [*p*_*FWE*_ < 0.001,k > 5 cm^3^] exclusively masked by IPU + *versus* IPU-). **b** Midline view of the bilateral brainstem cluster. **c** Central sulcus and **d** dorsal cerebellum. Bar graphs provide ß estimates for the left (L) and right (R) clusters maxima in the Resp- (−) and Resp+ (+) conditions. The present figure, including the 3D coordinate axes (X, Y, Z), was created by the authors using the CONN toolbox and does not infringe on any copyright.

Whole population contrast Resp + *versus* Resp- (*p*_*FWE*_ < 0.05, extend k > 5 cm^3^) exclusively masked with the contrast IPU + *versus* IPU- (*p*_*FWE*_ < 0.001) revealed bilateral activations in the central sulcus, brainstem and dorsal and ventral cerebellum (see Table 1; Fig. 3). Note that the reverse contrast revealed no activations at the thresholds used. The attribution of cerebellum clusters to the primary fissure and hemispheric lobule VIII for the dorsal and ventral clusters, respectively, relies on a cerebellum atlas, allowing the use of the MNI coordinate system with visual inspection of the cluster with respect to cerebellar folia boundaries^37^.

**Table 1:** Significant activations associated with the contrast Resp + ***versus*** Resp- (***p***_***FWE***_ < 0.001, extend > 5 cm^3^). MNI coordinates (in mm) are used to locate the clusters of significantly increased activity. *indicates that the clusters span the two hemispheres, making it impossible to attribute an extent to each of the two hemispheres.

Location		Extent (voxels)	Peak T values	MNI coordinates (mm)		
Anatomy	Side			x	y	z
Central Sulcus (fundus, dorsal part)	R	819	16.13	20	−27	60
	L	681	15.28	−21	−30	63
Cerebellum (centred on Primary Fissure)	R	5676*	21.36	14	−60	−20
	L		20.96	−15	−60	−18
Brainstem (Ventral Respiratory Column)	L	706*	10.82	−8	−38	−45
	R		10.47	8	−38	−45
Cerebellum (hemispheric Lobule VIII)	L	763	14.38	−12	−66	−50
	R	1141	14.16	10	−68	−48

**Fig. 4:**
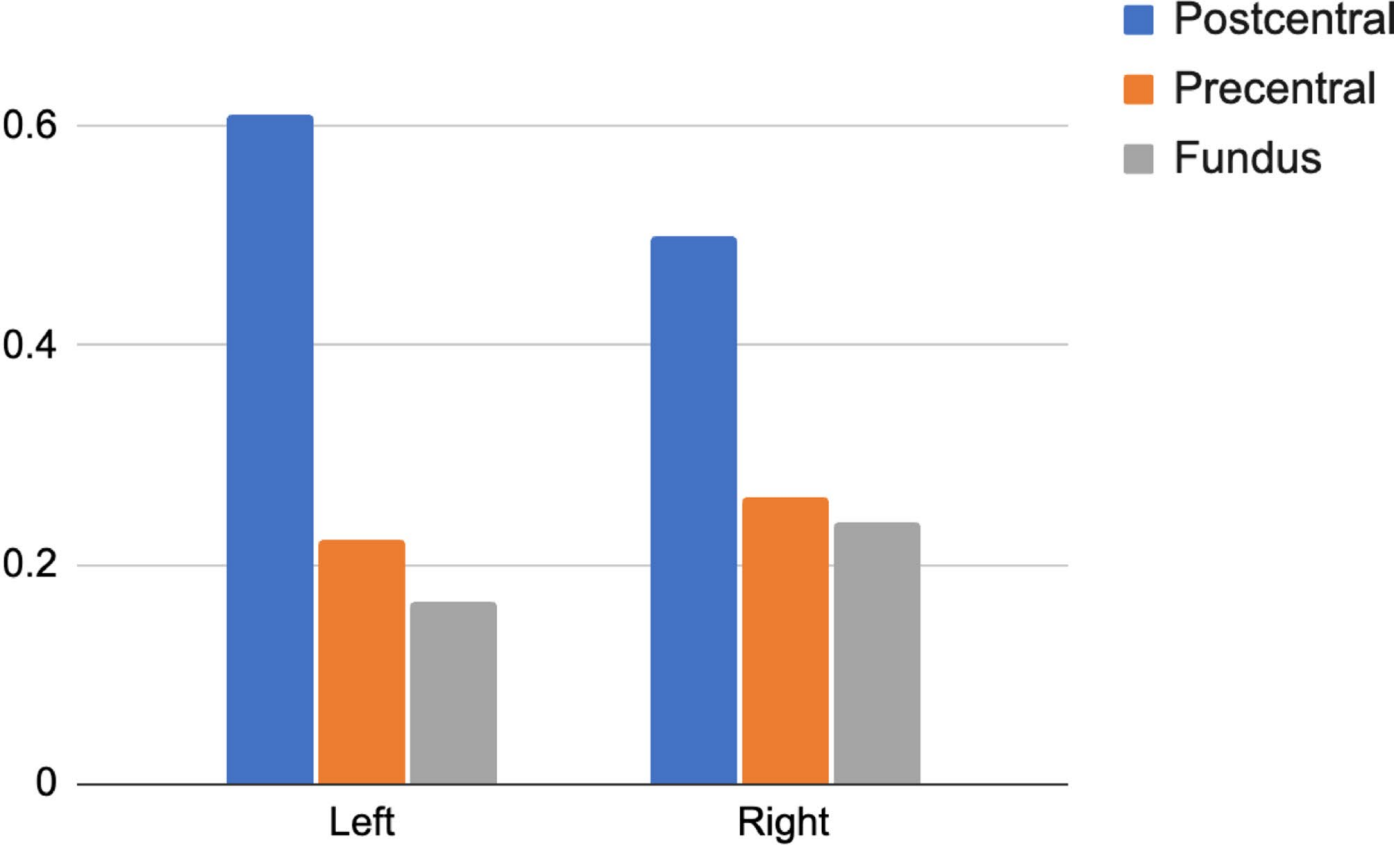
Individual participants and sessions contrast maxima in Resp + *versus* Resp- (*p*_*unc*_ < 0.001), within a 1-cm radius sphere centred on the group central cluster peak of activity, were semiautomatically attributed to the fundus of the sulcus and the pre- and the postcentral gyri. The figure presents the number of observations for the three possible cortical assignments.

The precise attribution of the respiration clusters to the pre- or post-central gyrus, triggered by the observation of Fig. 3 (3b), was addressed directly at the single participant and session levels, and the results are illustrated in Fig. 4 (single participant and session images are provided in Supplementary Fig. 3). Although these results suggest that clusters of maxima are mainly found in the postcentral gyrus, a comparable number of maxima were attributed to the anterior or fundus region of the central sulcus. However, anatomical (the fundus of the sulcus) and functional (transition from primary sensory to primary motor cortices) regions do not match, and the cytoarchitectonic boundary can extend towards the anterior precentral gyrus (see^38^). By extrapolation, considering that all postcentral gyrus clusters should be attributed to the primary sensory cortex and that even a fraction of the clusters associated with the other cortical regions could also belong to this functional area, we propose that bilateral central sulcus activity reflects a neural correlate of sensory processing related to Inspiration-to-Expiration transitions.

Finally, the localisation of functional areas in the brainstem is particularly challenging given the small size of brainstem nuclei compared with the fMRI voxel size, uncertainties regarding interindividual variability and the absence of a reference atlas. A postmortem brainstem atlas indicated that the brainstem cluster is located between the cerebellocortical and thalamocortical bundles identified via tractography^39^. Circumscribed by white fiber tracts and spanning the medullary and pontine regions, this bilateral cluster, which is elongated along the main axis of the brainstem and is characterised by maximum respiration events, likely belongs to the human ventral reticular formation, which is located in the homologue of the VRC identified in rodents^26^. More precise attribution of the activated cluster to a single brainstem nucleus would be merely speculative given current literature.

## Discussion

The present study aimed to investigate the neurophysiological underpinnings of speech-related respiration events anticipating conversational turn-taking. We analysed an existing corpus of natural conversations between a participant and its interlocutor (human or robot), focusing on synchronised (1) behavioural (conversation turn-taking), (2) respiratory (maxima of inspiration and expiration), and (3) neurophysiological (fMRI) recordings. Each participant’s speech production during the conversation was recorded, denoised, and segmented into silences and speech turns. The participants’ speech turns were defined as Inter Pausal Units -IPUs- i.e., blocks of continuous speech blocks occurring between silences lasting a minimum of 200 ms^35^.

Contrary to our initial expectation that Resp + events would be linked to high-level cortical mechanisms involved in turn-taking, our findings predominantly highlight neural circuits responsible for the control of respiration. Rather than reflecting the cognitive anticipation of speech in turn-taking, the observed activations in the brainstem, central sulcus, and cerebellum suggest a network specifically dedicated to regulating breathing for preparing speech production during a conversation.

### Respiration maximum and speech onset

We first investigated the temporal relationship between respiration maxima and speech production. The closest respiration maximum, in absolute value, was associated with the onset of each IPU and hence referred to as “Respiration maxima associated with IPUs” or “Resp+”. We further investigated the distribution of temporal associations between expiration and vocal production by focusing on the relative time differences between the two categories of events. As illustrated in Fig. 2, the cumulative density plot across all participants, sessions, trials, and turns indicated that maximum respiratory intake occurred, on average, 200 ms before speech onset. Variability can be due to the accumulation of temporal noise, both from the fMRI noisy recording environment precluding millisecond precision in the identification of the participant’s speech onset as well as from the saturation of the recording belt signal, which occasionally requires interpolation causing uncertainties in the precise timing of the respiratory maxima. Nevertheless, Supplementary Fig. 2 shows that the blue section of the density plots left to 0 ms (interval [−500; 0] ms) has the highest number of observations for most participants and recording sessions.

This result aligns with studies indicating a temporal delay between the end of air intake (respiration maximum) and the speech onset of approximately 200 ms^40^. Other studies have considered various factors. For example, one study reported that inhalation depth correlates with the speech envelope’s total power and is related to upcoming speech, suggesting internal models for enhanced communication^41^. Another study highlighted that respiration and pauses are crucial for turn-taking, showing that successful turns often occur immediately after a new inhalation, indicating that speakers coordinate their breathing with turn-taking^3^.

Taken together, the temporal relationship between speech and respiration appears to be preserved despite the lack of naturalness experienced when speaking while lying supine in a scanner during noisy fMRI acquisition. For the fMRI analysis, we defined “Resp-” events as “Respiration maxima not associated with IPUs”. Together, Resp + and Resp- identify all respiration maxima and are used as events for the fMRI analysis. Note that the physiological data (cardiac and respiratory cycles) were processed via the PhysIO Toolbox^34^ and used to derive nuisance variables describing noise induced by heart pulsation, respiratory volume and individual participants’ movements derived from functional magnetic resonance image (fMRI) realignment. Physiological artefacts are removed with this denoising procedure, and the results obtained reflect central nervous system respiratory control associated with conversational speech onset and not artefacts derived from the influence of peripheral physiology on brain recordings^42^.

### Speech-associated respiration maxima and central sulcus response

Several regions around the central sulcus are involved in modulating respiratory activity. Studies have shown that these specific regions are activated during the initiation and coordination of voluntary respiratory movements by sending signals to brainstem structures involved in regulating respiratory rhythms, such as the inferior olive bilaterally^43^. The central sulcus is also involved in speech production. Specifically, the primary motor cortex is responsible for the control of the muscles necessary for producing speech sounds. It sends signals that are transmitted through regions of the brainstem (though the exact pathways remain elusive, see^44^) to control precise movements of the laryngeal muscles, vocal folds, and articulators involved in speech. Motor cortices are therefore essential in coordinating and regulating speech-producing movements.

The exact location of the cluster at the fundus of the central sulcus (see Fig. 2) made it difficult to attribute the response across participants to the pre- or postcentral gyri, especially given that the boundary between cytoarchitectonic Brodmann areas 3 (primary somatosensory cortex in the postcentral gyrus) and 4 (primary motor cortex in the precentral gyrus) is at the fundus of the central sulcus^38^. Given that the data smoothing associated with DARTEL normalisation and group-level analysis could explain the impossibility of a clear attribution, we analysed the localisation of the cluster in single participants and sessions with unsmoothed anatomical and functional data in a semiautomatic fashion. The results showing the relative position of individual participants’ cluster of activity in the central sulcus drawn (shown in Supplementary Fig. 3 and summarised in Fig. 4) reveal that a majority of activated clusters for the contrast Resp + *versus* Resp- are found in the postcentral gyrus. As this would locate them in primary sensory cortices, the central sulcus cluster would be related to sensory processing and not motor control. These results suggest that sensory information from the chest upper body area, for example, the diaphragmatic aspects of respiratory functions, could be processed in this primary sensory region. This anatomical ambiguity also limits the possibility of clearly distinguishing between dorsal and ventral motor cortical contributions as described in previous models of respiratory versus articulatory control^2,10–13^. While our findings are consistent with somatosensory processing of respiratory-related input in the postcentral gyrus, the resolution of our data does not allow us to disentangle whether motor signals from dorsal premotor regions associated with respiratory coordination were also present. This interpretation aligns with previous research demonstrating that the postcentral gyrus contains somatosensory representations of respiratory-related body regions, including the chest and upper torso^45^. Given that respiration involves dynamic sensory feedback from the diaphragm, intercostal muscles, and upper chest region to maintain appropriate speech-breathing patterns, it is plausible that the observed cluster, in a dorsal position within the postcentral gyrus, reflects sensory monitoring of these regions during speech breathing.

### Speech-associated respiration maximum response in the brainstem

This sensory component found in the central sulcus might be associated with “perceiving” the maximum oxygen intake in the lungs required for producing subsequent speech. This could in turn also explain the increased response to Resp + events in brainstem regions associated with the control of breathing. The cluster was attributed to the VRC, which contains both inspiration (preBötC) and expiration neurons (PiCo). This complex has multiple subcortical, but very limited cortical, projections in rodents^46^.

The main contribution of the VRC is to generate rhythmic respiratory patterns^26^. Studies suggest that connections indeed exist between the motor cortex and brainstem regions involved in respiratory regulation, including the preBötzinger complex, as well as other interconnected pontine and medullary nuclei, such as the nucleus ambiguus, the inferior olive or the periaqueductal gray matter, which are also involved in vocal production^47^. These connections enable bidirectional communication between the cortex and the brainstem to coordinate the respiratory movements necessary for speech production^44^. These interactions may occur through descending projections from the motor cortex to the brainstem, regulating VRC activity and influencing respiratory control. Reciprocally, signals from several brainstem nuclei project back to the cortex and can carry sensory information about the current phase of the respiratory cycle given the level of inspiration^48^.

While the cluster’s location found in the current study is consistent with regions involved in respiratory control, particularly the VRC, future research using higher-resolution neuroimaging methods or complementary techniques, such as brainstem-targeted fMRI or electrophysiological recordings, may provide more direct evidence of the VRC’s functional role in speech-breathing processes.

### Speech-associated respiration maxima and cerebellar response

This temporal dynamic is reflected in the third result of the fMRI analysis, which identified significant activation in cerebellar clusters located in the bilateral primary fissure (lobules V and VI) and the right hemispheric lobules VIIIa and VIIIb, with greater activation for the Resp+ condition. The cerebellum, a brain structure traditionally associated with motor coordination and balance, has increasingly drawn attention for its significant role in speech production and perception^49^. These results suggest that the cerebellum plays a crucial role in regulating the speech rate, with evidence indicating its involvement in increasing speech rates beyond 3 Hz, emphasising its importance in the temporal organisation of verbal utterances. In addition, damage to cerebellar fibre tracts in lobule VI may further compromise speech motor control, contributing to ataxic dysarthria, a speech disorder characterised by difficulty in articulation and pronunciation^50^. Other studies highlight the functional convergence of motor and social processes in lobule IV/V, suggesting a broader role in cognitive and social behaviours^51^. Finally, lobule VIII is implicated in language processing, with studies reporting correlations between greater grey matter volume in lobules VII and VIII and better performance on measures of language^52^. Importantly, one function repeatedly associated with the cerebellum is the timing associated with motor control^53^. Given the importance of the relative timing between the events under investigation—the maximum respiration intake and the initiation of speech—it is proposed that the cerebellar responses observed in association with maximum respiratory events reflect preparatory components of vocal production. Moreover, although not classically defined as a respiratory structure, recent anatomical and physiological evidence suggests that the cerebellum contributes to respiratory regulation through reciprocal connections with brainstem respiratory centres, including the ventral respiratory group, and is actively engaged during chemical and mechanical respiratory challenges, such as hypercapnia and tracheal occlusion^54–57^.

### A cortico-brainstem-cerebellar network for speech turn-taking in natural conversation

We identified three key brain regions involved in the coordination between the respiratory maximum and speech onset during natural conversation: the postcentral sulcus dorsally, possibly involved in detecting sensory information from the upper airways; a brainstem region most likely associated with the respiratory pattern generators in humans; and cerebellar regions that could be involved in the temporal coordination of motor control. The increased response in the brainstem cluster, which is supposedly involved in the generation of breathing rhythms, likely reflects the active inhibition of the respiratory pattern generators that are required to adapt expiration to vocal production. Had inhibition of brainstem respiratory pattern generators been passive, we would have expected an increased response in the brainstem in the opposite contrast focusing on maximum respiratory intake not associated with speech production, which yielded no significant response in the current analysis. The source of this inhibition could be the cerebellum clusters found in the same contrast, as direct inhibition of respiration by electric stimulation of Purkinje cells has been reported (reviewed in^58^. This interpretation is in agreement with the role of the cerebellum in the temporal aspects of motor control. Finally, the central sulcus cluster, attributed to the primary sensory cortex, can be activated by afferents from upper airways effectors, carrying the information that the lungs are full and therefore ready to initiate speech. This important piece of information could also be integrated by brainstem and/or cerebellar components.

These findings are consistent with well-established fMRI studies in healthy individuals showing that voluntary respiratory tasks—such as hyperpnea and sniffing—activate a broad cortical network including the supplementary motor area, sensorimotor cortex, and premotor regions, alongside subcortical structures like the medulla^2,59^. Moreover, a comprehensive recent review of central respiratory control in humans—including studies using CO₂ inhalation, hypercapnia, breath-holding, and induced hypoxia—supports the engagement of both brainstem rhythm generators and suprapontine cortical regions in healthy volitional breathing control^60^. Such evidence underscores that respiration during speech is not an isolated motor event but shares neural substrates with other forms of voluntary breathing.

Additional support is provided by clinical evidence. For example, hyperpnea, which shares certain neural activations with speech breathing, is characterised by strong activation of the supplementary motor cortex, the precentral gyrus, and the left sensorimotor cortex, with bilateral enhancement in the medulla^2^. Similarly, sniffing activates the sensorimotor, premotor, and supplementary motor cortices and the motor area of the cingulate cortex, suggesting the inhibition of medullary respiratory centres during breath retention^59^ and a complex interplay of brain regions in the modulation of breathing during speech. Although speech appears to be a particular respiratory control event, these correlative data suggest that attention to brain activation during speech is needed. A study^15^ confirmed the role of the cortical component in respiratory preparation during verbal reproduction, with excitatory and inhibitory modulations of diaphragm control by the supplementary motor cortex. The authors suggest the inhibition of automatic inspiration during utterance, potentially by the corticobulbar tract involved in voluntary breath retention, with breath-specific inhibitory commands playing a role in respiratory control during speech. These results suggest that both cortical and subcortical brain regions are involved in the control of breathing during turn-taking associated with natural conversation.

The absence of significant activations in higher-order cortical areas may be due to power limitations inherent to our fMRI design, as isolating speech intention from respiration signals presents methodological challenges. However, this does not diminish the relevance of our findings. The observed activations in brainstem and cerebellar structures highlight the precise sensorimotor regulation of breathing during conversational speech onset. Taken together, the relatively complex synchronisation of breathing with speech production involves the combination of several pieces of information reflected in the activation of brain areas belonging to different regions of the central nervous system.

## Conclusion

Our study confirmed a temporal relation between respiration and conversational dynamics and identified several key actors involved in this relation at different levels of the central nervous system. These actors are the dorsal central sulcus, possibly corresponding to the primary sensory cortex encoding information coming from the chest level that can be associated with the filling of the lungs; the cerebellum, likely involved in computing the temporal dynamics between lung filling and speech onset; and the brainstem, where respiratory rhythm generators of the VRC must be actively inhibited, consistent with established principles of cortical inhibitory mechanisms in the literature, to entrain respiration to speech production. While speculative, these interpretations are based on a large but scattered results gathered from literature that investigated the different actors in isolation or by pairs, especially in animal studies, complemented by the neuropsychological results of speech impairments in humans. However, to our knowledge, this is the first time that a corpus of natural conversations by humans, including synchronised functional neuroimaging, is used to identify the actors involved in breathing and speech turn-taking. While further research will be necessary to investigate the dynamics of this central nervous system network, these results underscore the importance of integrating peripheral physiological signals to investigate complex social behaviours in ecological settings.

## Supplementary Information

Below is the link to the electronic supplementary material.

Supplementary Material 1

Supplementary Figure 2

## Data Availability

The datasets analysed during the current study are available in ORTOLANG at the present ink: https://www.ortolang.fr/market/corpora/convers/v2 (linguistic data) and in OpenNeuro repository: https://openneuro.org/datasets/ds001740/versions/2.2.0 (fMRI raw data).
