# Supplementary material for: Central nervous system control of breathing in natural conversation turn-taking
Source: Sci Rep. 2025 Aug 25;15:31276. doi: 10.1038/s41598-025-15776-1 (PMC12378384; doi:10.1038/s41598-025-15776-1)
Supplement: Supplementary file 1 — Supplementary Material 1 [file 41598_2025_15776_MOESM1_ESM.docx]

**Supplementary Tables and figures**

**
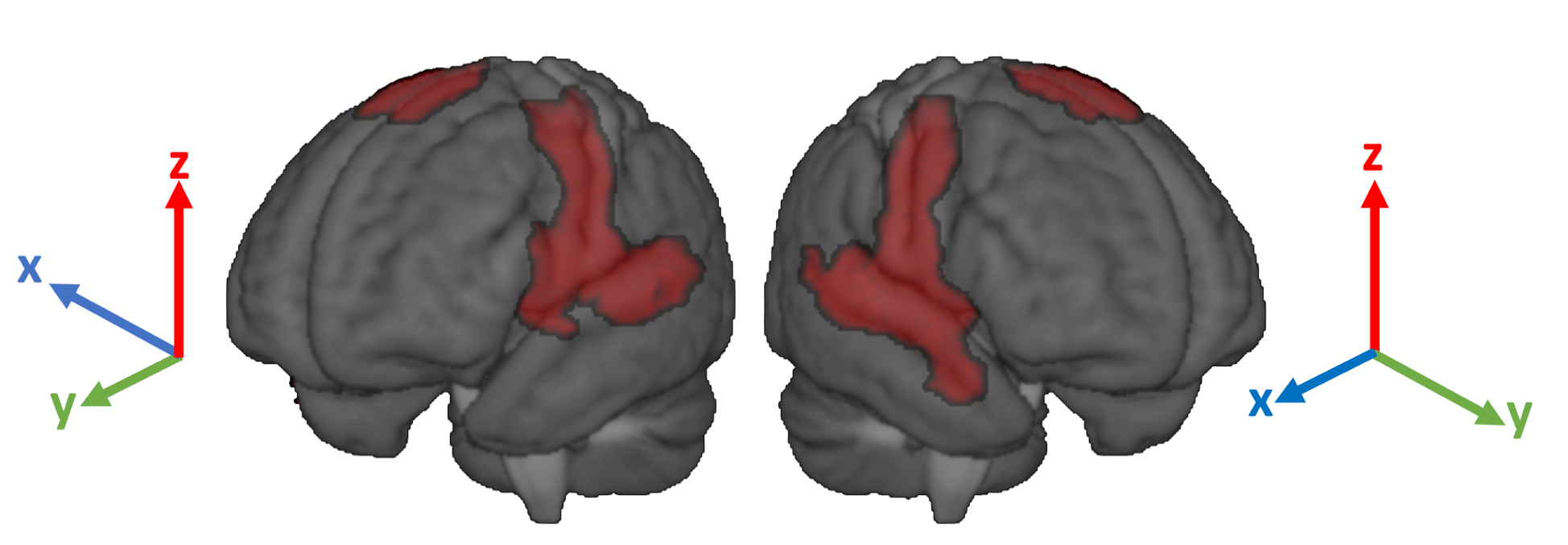
**

**Supplementary Figure 1: Contrast between IPU⁺ and IPU⁻ events.** Brain regions exhibiting increased activation during speech segments (IPU+) compared to those non-speech (IPU-). This contrast was used as an exclusion mask in the main analysis (Resp+ vs Resp-) to remove activity related to speech production. The identified network involves bilateral motor and auditory cortices, the left inferior frontal gyrus (IFG), and medial premotor areas (SMA and preSMA). Subcortical engagement of the anterior putamen was also observed, though not visible in this surface rendering. The present figure, including the 3D coordinate axes (X, Y, Z), was created by the authors using the CONN toolbox and does not infringe on any copyright.

**Supplementary Figure** **2: Single participant and run density plots of Δt providing the mean and skewness of the distribution.**


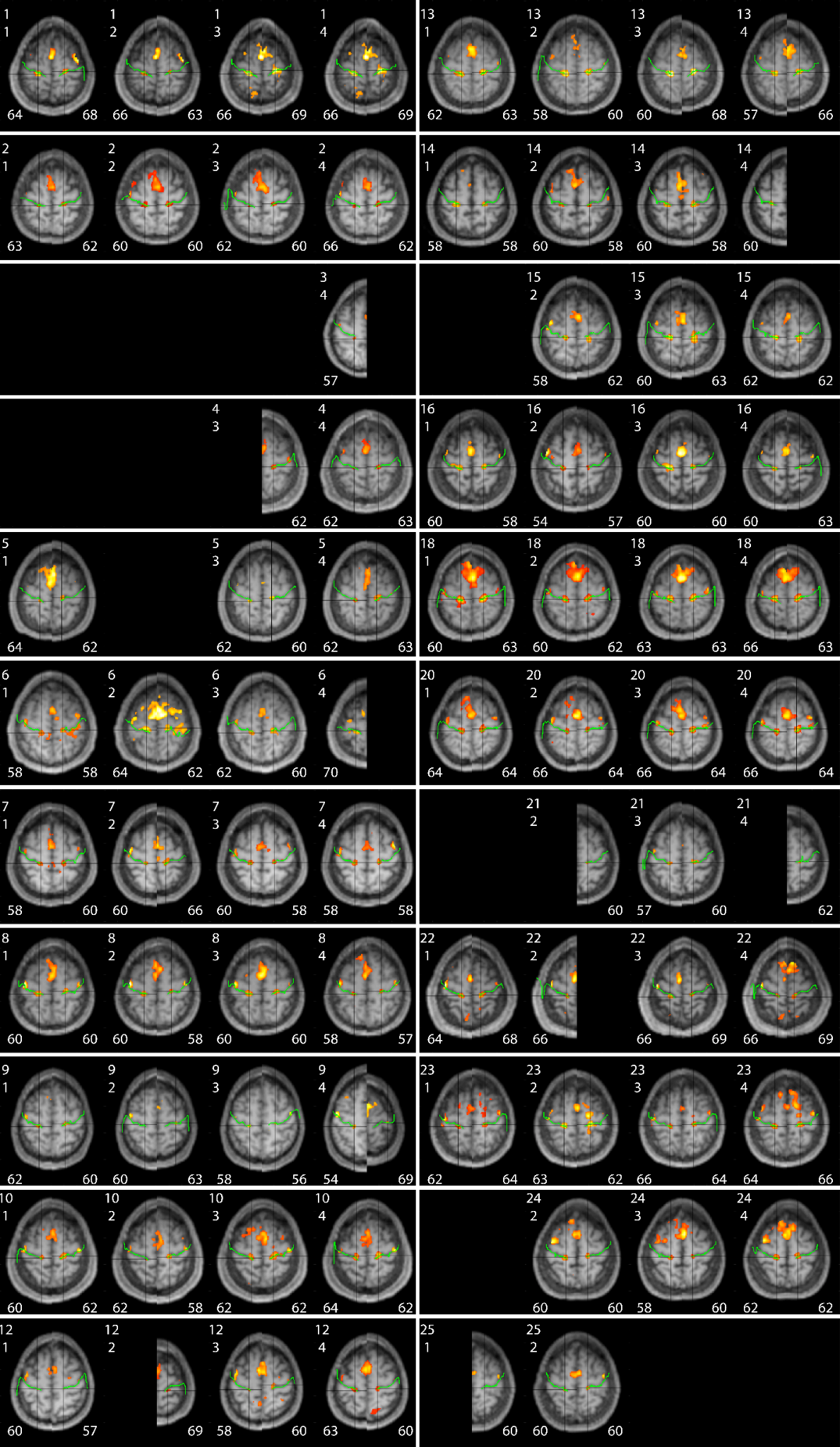


**Supplementary Figure 3: Single participant and run analysis of the relative location of the central sulcus respiration cluster with an automatic tracing of the central sulcus in green.**

**
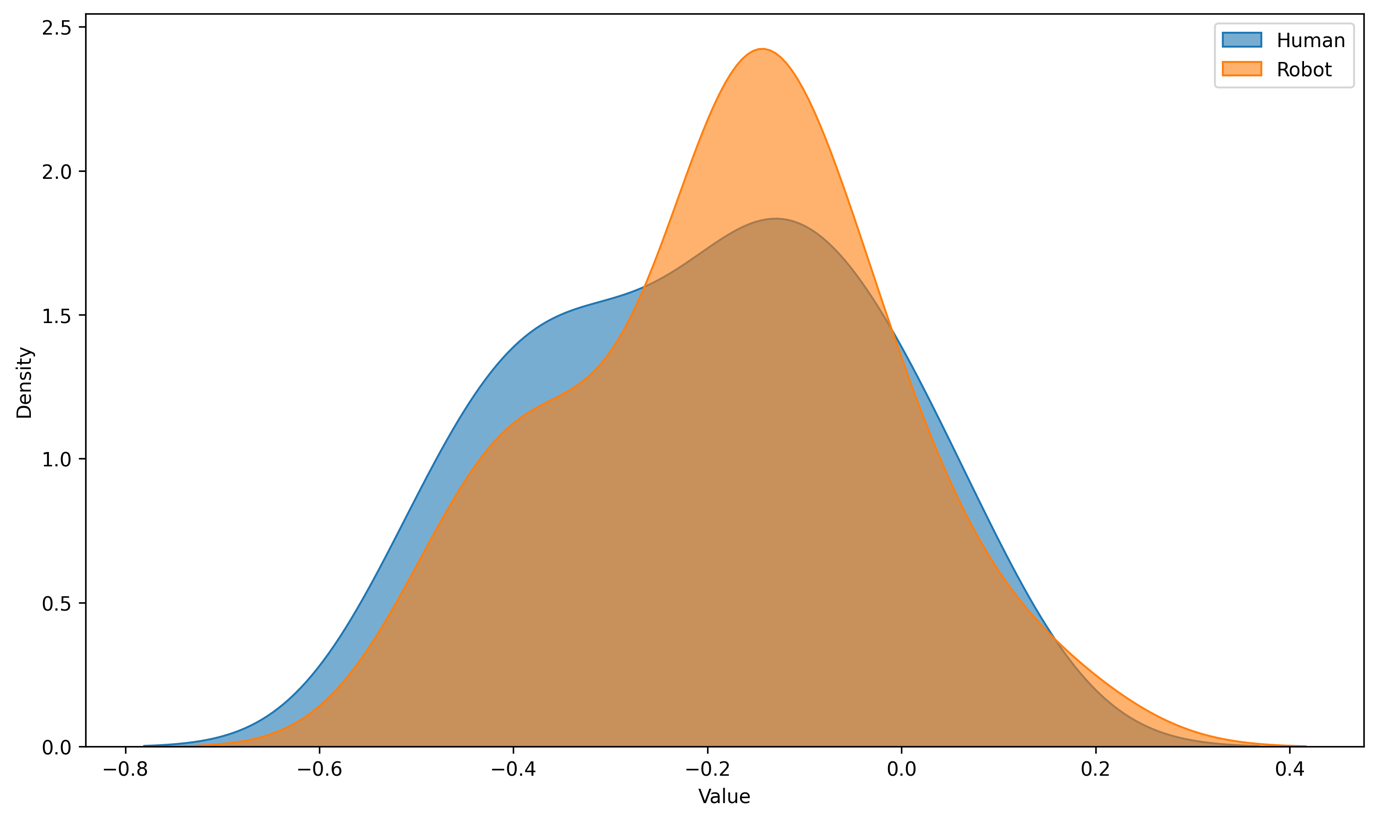
**

**Supplementary Figure 4: Distribution of Resp+ ∆t values for human (blue) and robot (orange) interlocutors.** The density plots illustrate the distributions for the two groups, both centred before the respiratory peak (mean Human: -0.2063 s, *s.d.* 0.1752 mean Robot: -0.1804 s, *s.d.* 0.1617).
