# Supplementary figures and images for: Central nervous system control of breathing in natural conversation turn-taking
Source: Sci Rep. 2025 Aug 25;15:31276. doi: 10.1038/s41598-025-15776-1 (PMC12378384; doi:10.1038/s41598-025-15776-1)

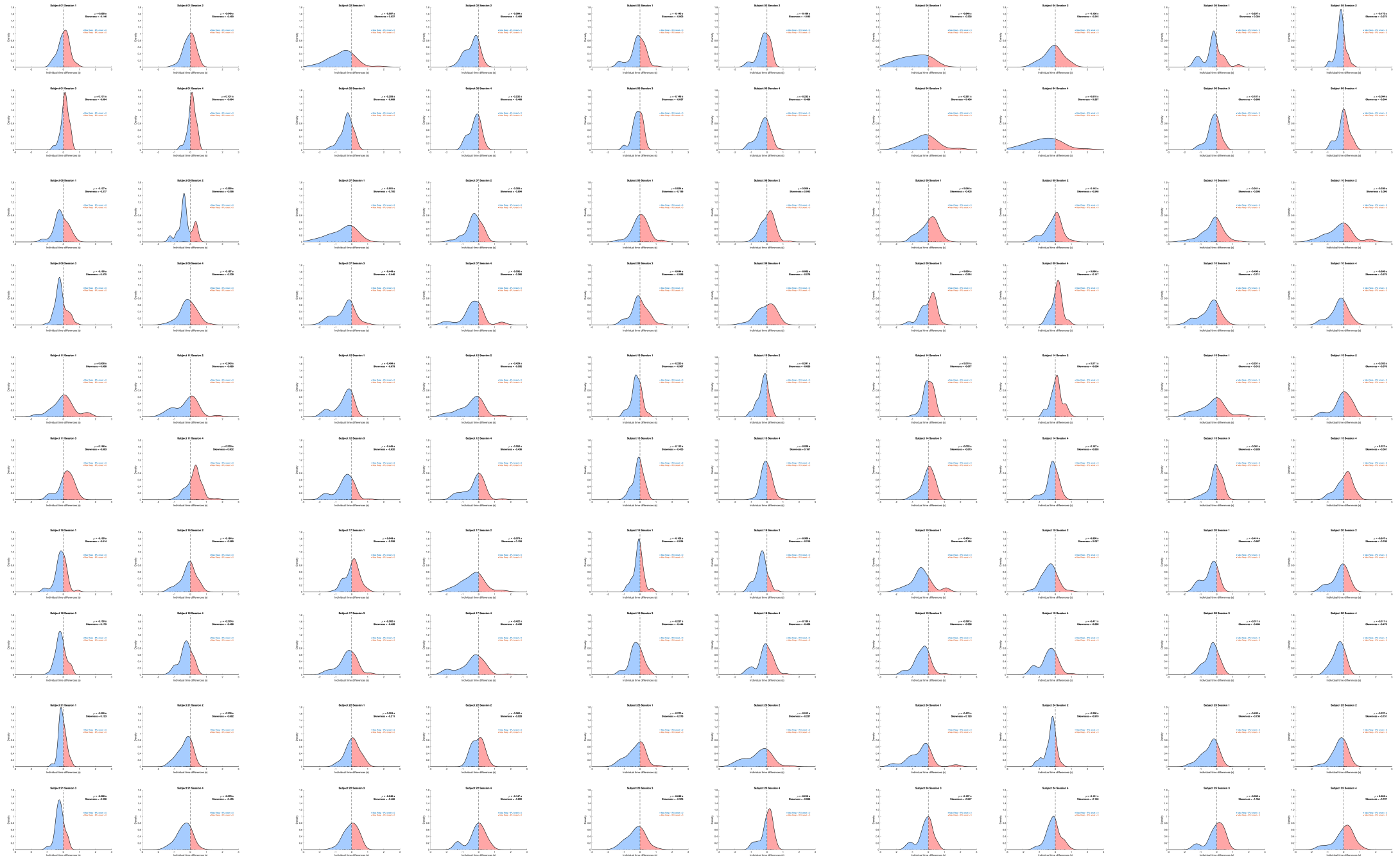

Supplement: Supplementary file 2 — Supplementary Figure 2 [file 41598_2025_15776_MOESM2_ESM.pdf]
